# Breaking Through Disciplinary Barriers: Human–Wildlife Interactions and Multispecies Ethnography

**DOI:** 10.1007/s10764-018-0027-9

**Published:** 2018-04-18

**Authors:** Hannah E. Parathian, Matthew R. McLennan, Catherine M. Hill, Amélia Frazão-Moreira, Kimberley J. Hockings

**Affiliations:** 1 Centre for Research in Anthropology (CRIA-FCSH/NOVA), 1069-061 Lisbon, Portugal; 2 Anthropology Centre for Conservation, Environment and Development, Department of Social Sciences, Oxford Brookes University, Oxford, OX3 0BP UK; 3 Bulindi Chimpanzee and Community Project, P.O. Box 245, Hoima, Uganda; 4 Department of Anthropology, Faculty of Social and Human Sciences, New University of Lisbon, Lisbon, 1069-061 Portugal; 5 Centre for Ecology and Conservation, University of Exeter, Penryn, Cornwall, TR10 9FE UK

**Keywords:** Conservation conflict, Ethnoprimatology, Human–wildlife conflict, Human–wildlife interactions, Interdisciplinary research, Multispecies ethnography, Primate conservation

## Abstract

One of the main challenges when integrating biological and social perspectives in primatology is overcoming interdisciplinary barriers. Unfamiliarity with subject-specific theory and language, distinct disciplinary-bound approaches to research, and academic boundaries aimed at “preserving the integrity” of subject disciplines can hinder developments in interdisciplinary research. With growing interest in how humans and other primates share landscapes, and recognition of the importance of combining biological and social information to do this effectively, the disparate use of terminology is becoming more evident. To tackle this problem, we dissect the meaning of what the biological sciences term studies in “human–wildlife conflict” or more recently “human–wildlife interactions” and compare it to what anthropology terms “multispecies ethnography.” In the biological sciences, human–wildlife interactions are the actions resulting from people and wild animals sharing landscapes and resources, with outcomes ranging from being beneficial or harmful to one or both species. In the social sciences, human–nonhuman relationships have been explored on a philosophical, analytical, and empirical level. Building on previous work, we advocate viewing landscapes through an interdisciplinary “multispecies lens” in which humans are observed as one of multiple organisms that interact with other species to shape and create environments. To illustrate these interconnections we use the case study of coexistence between people of the Nalu ethnic group and Critically Endangered western chimpanzees (*Pan troglodytes* *verus*) at Cantanhez National Park in Guinea-Bissau, to demonstrate how biological and social research approaches can be complementary and can inform conservation initiatives at the human–primate interface. Finally, we discuss how combining perspectives from ethnoprimatology with those from multispecies ethnography can advance the study of ethnoprimatology to aid productive discourse and enhance future interdisciplinary research.

Humans have presumably coexisted with nonhuman primates (hereafter primates) throughout our evolution, yet there can be little doubt that today humans and primates share landscapes to an unprecedented extent (Humle and Hill 2016; McKinney 2015; Paterson and Wallis 2005). Identifying strategies to overcome constraints to sustainable coexistence must become a priority for conservation if primates are to survive the Anthropocene (the current geological epoch of human dominance of geological, biological, and chemical processes on Earth, usually dating from 1945 in ecology and conservation; Corlett 2015) (Estrada *et al.* 2017; Fuentes and Wolfe 2002; Hockings *et al.* 2015; McLennan *et al.* 2017). To develop effective, locally appropriate strategies to conserve primates and other wildlife, it is essential to understand human social and cultural variables alongside wildlife behavioral and population patterns. This requires a combination of social science and biological science methods of inquiry (Bennett *et al.* 2017a, b; Dore *et al.* 2017; Jost Robinson and Remis 2014; Mascia *et al.* 2003; Redford 2011; Setchell *et al.* 2017; Wolverton *et al.* 2014). Conservation biology increasingly engages with social science, including anthropology, sociology, political ecology, and psychology (Daily and Ehrlich 1999; Mascia *et al.* 2003; Newing 2010; Teel *et al.* 2018), yet interdisciplinary barriers to communication can hinder development of productive discourse (Bennett *et al.* 2017b; Decker *et al.* 1987; Fox *et al.* 2006; Fuentes 2006). Potential collaborations are restricted through disparate academic terminologies and use of vocabulary often understood only by those with subject-specific knowledge (Moon and Blackman 2014). In the biological sciences humans are considered part of nature in an evolutionary sense, but are traditionally viewed as separate from nature in an ecological sense (Sponsel 1997). In accordance with this perspective, until recently primatologists and other biological scientists interested in the adaptive significance of behaviors sought to study animals in so-called “natural” environments, supposedly free of human influence. Consequently, there was less interest in the bidirectional interactions between people and wild animals, despite the fact that humans have long been a part of most ecosystems where primates and other wildlife are studied (Hockings *et al.* 2015; Riley 2006; Tutin and Oslisly 1995). In contrast, social scientists among other scholars in the humanities (e.g., Gillespie and Collard 2015; Keil 2016; Rose *et al.* 2012; Wilkie 2015) have considered the multiple possible realities perceived by diverse human communities and individuals that are shaped by religious and cultural beliefs, historical and social backgrounds, and ontological reasoning. Among human societies, people’s associations with wildlife range from ambiguous species boundaries and holistic concepts of nature that unite people, plants, animals, and supernatural beings to much more dualistic understandings whereby humans and animals, including primates, are considered as very separate entities, occupying distinct spaces (Aisher 2007; Aisher and Damodaran 2016).

Ethnoprimatology has taken steps toward combining social and biological science approaches to develop a more holistic understanding of primate ecology and conservation (e.g., Fuentes 2010a, 2012; Hardin and Remis 2006; Jost Robinson and Remis 2014; Malone *et al.* 2014; Remis and Hardin 2009; Remis and Jost Robinson 2017; Riley 2006, 2013; Sponsel 1997), but disciplinary barriers persist. To tackle this problem, we examine differences in the meanings of some commonly used terminology in the biological and social sciences. Specifically, we dissect the meaning of what the biological sciences (including primatology) term studies in “human–wildlife conflict” or more recently “human–wildlife interactions” (e.g., Hockings 2016; Humle and Hill 2016; Woodroffe *et al.* 2005) and compare it to what the social sciences term “multispecies ethnography” (e.g., Haraway 2008; Kirksey and Helmreich 2010). Using examples from ethnoprimatology we highlight the “distinct” approaches of biological and social science and discuss how combining them can enhance our understanding of shared landscapes and advance research at the human–primate interface. As we demonstrate using research examples in the text that follows, the distinction between these two approaches is increasingly blurred as biologically trained scientists seek qualitative nuance, and as socially trained scientists seek quantitative data on the nonhuman agents living among and influencing the behavior and lives of their human neighbors (Rust *et al.* 2017). We then critically interrogate a number of key concepts and advocate for an integration of multispecies approaches with ethnoprimatology (Fuentes 2010a; Malone *et al.* 2014; Palmer and Malone 2018; Remis and Jost Robinson 2017). Finally, we illustrate these links using a case study of coexistence between people of the Nalu ethnic group and Critically Endangered western chimpanzees (*Pan troglodytes verus*) at Cantanhez National Park in Guinea-Bissau, West Africa, to demonstrate how disciplinary theories, descending from biological and social science, can be combined and applied practically through interdisciplinary research approaches.

## Biological Sciences: Recognizing the Value of Social Science to Conservation

Biological approaches to understanding human–wildlife interactions and ecological relationships are grounded in the disciplines of behavioral ecology and conservation biology, originally the domain of ecologists and zoologists. Behavioral ecology is concerned mainly with the causes, evolution, and adaptive variation in behavior of individuals, whereas conservation biology has an interest in populations, especially their response to disturbance or environmental changes caused by humans (Caro and Eadie 2005; Sih *et al.* 2011). Using principles from ecology, population genetics, and systematics, conservation biology seeks to describe biological diversity and identify ways to conserve species and ecosystems (Mascia *et al.* 2003; Simberloff 1988). In the biological and conservation sciences, there has been a predominant focus on the ways in which wildlife “conflict” with the interests of humans (Angelici 2016; Messmer 2009; Paterson and Wallis 2005; Treves and Karanth 2003), often with a goal to identify general “large-scale” trends in the nature of interactions (e.g., Inskip and Zimmerman 2009; Seoraj-Pillai and Pillay 2017). For the last 20 years or so some primatologists (especially those who received their training within Anthropology departments), and some biologists, have approached human–wildlife interactions as dynamic and bidirectional (e.g., Fuentes and Wolfe 2002; Humle and Hill 2016; Hurn 2017; McLennan *et al.* 2017; Redpath *et al.* 2013; Wheatley 1999), though this remains a minority approach. While identifying large-scale trends is important, “small-scale” site-specific data are also needed to fully understand the diverse ways in which humans and wildlife interact in shared heterogeneous landscapes (Hockings 2016). Today, conservationists increasingly recognize that the success of conservation policies and practice inherently depends on understanding and addressing human social phenomena (Bennett *et al.* 2017b; Berkes 2004; Redford 2011; Redpath *et al.* 2013), and where conservation interventions pay inadequate attention to social factors they fail to conserve target species and ecosystems (e.g., Agrawal and Redford 2006; McLennan and Hill 2013; Rönnbäck *et al.* 2003; Rust *et al.* 2016).

Social science disciplines include subjects such as anthropology, psychology, sociology, politics, and international studies, and therefore have analytical tools that explain and predict patterns of human behavior and attempt to find meaning behind cultural or subjective phenomena. These offer unique and important insights into a given society’s understanding of their associations with wildlife, which has strong relevance for conservation practice and outcomes (Mascia *et al.* 2003). For example, social and cultural anthropology methods of inquiry can document the spiritual value of biodiversity to people. In primatology, this can be applied to identify conservation-relevant cultural beliefs and values that serve as foundations for formal regulations that protect primate species and habitats, or help guide locally appropriate conservation initiatives (Baker *et al.* 2014; Etiendem *et al.* 2011; Remis and Hardin 2009; Jones *et al.* 2008; Jost Robinson and Remis 2014; Köhler 2005; Wheatley 1999; Yamakoshi and Leblan 2013). While government and nongovernmental organizations (NGOs) increasingly take steps to integrate social science information into conservation decision making and long-term environmental management, success is still hampered by economic and institutional challenges. These include conflicts between stakeholders, inadequate financial support for local monitoring and governance (Sandker *et al.* 2009), and legal frameworks (specifically tenure and economic laws) that can present significant constraints to the longevity of such interventions (Pasquini *et al.* 2011). Barriers to effective collaboration and understanding between social and biological scientists and conservation practitioners further impede these developments (Fox *et al.* 2006).

## Social Sciences: Embracing the Complexity of Human–Animal Relationships

Anthropologists have repeatedly challenged environmental discourse that oversimplifies the complex relationships between humans and nonhuman species (e.g., Atran 1999; Descola 1994; Ingold 2000; Kohn 2007, 2013). Some of the earliest scientific studies addressing the intersection between biology, culture, and sociality originate in the discipline of ethnobiology. Ethnobiology encompasses botany, zoology, and ecology, and is broadly defined as the study of how living things are treated or used by different human cultures (Ellen 2006). While ethnobiology once focused largely on studies of folk classification (Bulmer 1967; Conklin 1954; Ellen 2006; Hunn 1977), today it is recognized essentially as the study of how people from different cultures conceptualize, represent, use, and manage their knowledge of environments and living organisms. As Ellen (2006, p. 3) suggests, “ethnobiology – like anthropology more broadly – seeks to go beyond the local, to compare such knowledge and its consequences between different human populations, and to establish generalizations that are valid at the regional, global, and species level.”

There has been a proliferation of interdisciplinary terms and fields of study by anthropologists, sociologists, and human geographers particularly, as they explore ways of incorporating nonhuman species into social science research. From Lestel’s “ecoanthropology and ethnobiology” (Lestel *et al.* 2006; Lestel and Taylor 2013) to Haraway’s (2010) “companion species,” researchers have endeavored to develop innovative frameworks for conceptualizing relationships between human and nonhuman species. For example, anthrozoology draws from various disciplines including anthropology, psychology, and zoology to examine human–animal relationships in relation to animal representations, symbols, and stories, and their physical presence in human societies (York and Mancus 2013). Meanwhile, zooanthropology explores relationship dynamics between humans and animals with a focus on animal sentience and well-being (Aerts *et al.* 2016; Marchesini 2016). As the name suggests, ethnoethology explores the methodological overlap of ethnology and ethology, examining the characteristics of different peoples and their relationships with animals and ecosystems (see Glossary for further examples). Early examples of interdisciplinary research in primatology include the work of Barbara Smuts and Shirley Strum, whose accounts of baboon groups in Tanzania and Kenya transgress the positivist norms of ethology as an observational science (Despret 2013; Smuts 2009; Strum 1987). Although these fields of study adopt differing perspectives, they offer useful methods for overcoming nature–culture duality and have been used to examine human–primate interactions and social representations of primates (for examples with African great apes: Giles-Vernick and Rupp 2006; Köhler 2005; Lingomo and Kimura 2009; Oishi 2013; Richards 1995; see also Jost Robinson and Remis 2014). Such studies provide insights into local understandings of nature that are highly relevant to establishing locally appropriate conservation practices. For example, interdisciplinary studies have revealed how Western-dominated ideals vs local perceptions of wildlife influence support, or lack thereof, for conservation (Jalais 2008), and how the choice of conservation flagship species needs to be appropriate to the target audience, taking into account local attitudes toward, beliefs about, and experience of local species (Sousa *et al.* 2018).

## Ethnoprimatology as an Interdisciplinary Study

Traditional Western primatology (compared to Japanese primatology; Asquith 1986 and de Waal 2001 provide comparisons of the two) has strived to adopt an objective view of the biological and psychological similarities between humans and primates. In contrast to the approach of traditional field primatology, ethnoprimatology aims to acquire an anthropological understanding of primates through examining their associations with human cultures and societies (Fuentes and Wolfe 2002; Papworth *et al.* 2013; Paterson and Wallis 2005; Sponsel 1997). Ethnoprimatological research employs mixed methods and embraces a multidisciplinary theoretical perspective to examine the multifarious interactions and interfaces at integrated and shared ecological and social spaces (Fuentes 2012; Hockings *et al.* 2015; Sponsel 1997). The goal of many ethnoprimatology studies is to engage with the needs of local human populations to enhance primate conservation and ensure the longevity of conservation projects by understanding the biological and social dynamics between humans and primates (Cormier 2010; Fuentes 2012; Jost Robinson and Remis 2014; Lee 2010; Malone *et al.* 2014; Papworth *et al.* 2013; Riley 2013; Wheatley 1999). The ethnoprimatological approach is described by Fuentes *et al.* (2017, p. 297) as “a mosaic of approaches that is developing, and reshaping, the ways in which humans position themselves relative to nonhuman primates (NHPs), and the ways in which NHPs are seen as agential in human-dominated landscapes, ecologies, and lifeways.” Social anthropologists have sought similar understandings of human–primate relationships. For example, ethnographic studies of traditional people’s understandings of African great apes incorporate local knowledge systems into conservation narratives (Etiendem *et al.* 2011; Giles-Vernick and Rupp 2006; Köhler 2005; Lingomo and Kimura 2009; Oishi 2013; Richards 1995). As with other interdisciplinary approaches discussed above, ethnoprimatology demonstrates an epistemological affinity between biological and sociocultural anthropology by acknowledging humans as active and integral members of biological communities (Leblan 2013; Riley 2006, 2010, 2013).

Growing enthusiasm for the ethnoprimatology approach, and recognition among conservation funding agencies that (for ethical and practical reasons) conservation in most instances is unsuccessful without integrating the needs of local people, has encouraged recent developments in primatology. The predominant emphasis on conflict and competition in studies of human–primate interactions (McLennan *et al.* 2017; Paterson and Wallis 2005) is gradually giving way to a greater appreciation of the complexities of these relationships, including “positive” interactions (Frank 2016). For example, research at Bossou in the Republic of Guinea showed how consumption of cultivated cocoa by western chimpanzees, and subsequent dispersal of seeds, led to the widespread distribution of cocoa plants in the habitat, benefitting both local farmers and chimpanzees (Hockings *et al.* 2017). In parallel, there have been calls for a linguistic shift in how human–primate interactions are framed and described (e.g., from “crop raiding” with its aggressive connotations to a more neutral “crop feeding” or “crop foraging”; Hill 2005, 2015, 2017; Hill *et al.* 2017). It is now broadly accepted that humans are key components of ecosystems where primates live (Fuentes and Wolfe 2002; Hockings *et al.* 2015; McKinney 2015; McLennan *et al.* 2017). Rather than viewing human communities and practices as uniformly damaging to natural habitats, the traditional methods that local people have used and adapted over millennia to manage and monitor landscapes are increasingly acknowledged as potentially useful foundations for developing practical conservation strategies (Berkes *et al.* 2000; Thompson *et al.* 2008; Yamakoshi and Leblan 2013).

Studies examining the social constituents of primate conservation have revealed that people’s views of primates are influenced by political, social, and economic factors, which are not fixed but change over time (e.g., Hill and Webber 2010 in Uganda; Parathian and Maldonado 2010 in the Colombian Amazon). Other studies demonstrate how unique belief systems and human–primate associations can support protection of primate species, for example, long-tailed macaques (*Macaca fascicularis*) in Bali: Fuentes *et al.* (2005) and Wheatley (1999); and Tonkean and booted macaques (*Macaca tonkeana* and *Macaca ochreata*) in Sulawesi: Riley (2007, 2008, 2010); Riley and Fuentes (2011); and Riley and Priston (2010). Similarly, a study by Etiendem and colleagues discusses how traditional totemic beliefs about Cross River gorillas (*Gorilla gorilla diehli*) in southwest Cameroon can be revived and promoted to foster positive attitudes to gorilla conservation (Etiendem *et al.* 2011). While significant progress has been made in the field of ethnoprimatology, further developments are essential in terms of primatologists adopting mixed epistemologies and methodologies. Moreover, until recently there have been few sources of funding available to provide graduate training to link disparate fields or offer financial support to projects that study complex interactions through interdisciplinary concepts and practice (Fuentes *et al.* 2017; Palsson *et al.* 2013).

## Barriers to Interdisciplinary Communication

Recognizing that conservation is as much about people as about other species and habitats requires significant modifications to how science is used and applied in conservation. Bennett *et al.* (2017b) outline major barriers to the meaningful integration of social science into conservation science, stemming from unfamiliarity with subject-specific principles, limited collaboration, and academic boundaries aimed at “preserving the integrity” of subject disciplines. Academic researchers are usually trained in traditional disciplines and may lack the tools or willingness to make bridges between fields. They may have differing “theories of knowledge,” including their philosophies, worldviews, and epistemologies, which can lead to incompatible ways of perceiving human–wildlife interactions or approaching research into these phenomena (Moon and Blackman 2014; Rust *et al.* 2017). For example, in a study exploring the environmental impacts of deforestation the social scientist may begin by talking to people in a local village to understand the effects on human behavior, while the natural scientist may begin by exploring ecological indicators (Bennett *et al.* 2017b). Furthermore, discipline-specific language and the different theories applied to understand particular topics can be inaccessible to nonspecialists or specialists in other subjects. Issues of familiarity with the diverse literature and associated nuances in language can present additional obstacles (Lemke 2001), while subject-specific discourse used by social scientists and biologists presents boundaries to cross-disciplinary collaboration. The language used by social scientists can be intentionally ambiguous, to reflect alternative worldviews of cultures that oppose Western dichotomized notions of nature (Descola 2014; Kohn 2007), and/or to challenge preconceived ideas and assumptions about the world that characterize a Western scientific approach. For these reasons, biological anthropologists trained to be objective, realist, and positivist in their research approach can find these concepts difficult to grasp. They may view social studies as too time-consuming (when conservation decisions often need to be made rapidly), or vague and “esoteric.” Conversely, social anthropologists tend to consider biological methods as overly pragmatic and rigid in their application (especially as real-world problems are complex). This can lead to important but not immediately visible information being overlooked. The core beliefs and ideas of these disciplines can appear so different that biological and social scientists have been said to come from different “academic *cultures*” (Morris 1969; Sutherland 1998; see Glossary). This may indeed be true, but as Kohn reminds us: “The goal [in multispecies ethnography] should not just be to give voice, agency or subjectivity to the nonhuman—to recognize them as others, visible in their difference—but to force us to radically rethink these categories of our analysis as they pertain to all beings [March 29, 2010]” (in Kirksey and Helmreich 2010, p. 563).

In biological anthropology sympatric species are viewed as individuals engaged in bidirectional dyadic relationships, which meet temporally or have some impact on each other’s lives, for example, by affecting the availability of certain resources or shaping forest habitats in certain ways. These “human–wildlife interactions” result in either positive or negative outcomes for one or both species. By comparison, in social anthropology humans and wildlife (including nonhuman organisms broadly) are considered as close companions (Haraway 2010, 2016), innately and immutably linked through complex ecological, historical, social, cultural, and political networks; for examples, see Locke’s (2013, 2017) exploration of human–elephant relations in Asia, and Jost Robinson and Remis’s (2014) analysis of the mutual ecologies of “the hunter and hunted” in Central Africa. These ideas describe the long-term mutual exchange and emergence of human and nonhuman companions including other primates.

In the social sciences the term “multispecies ethnography” refers to a methodological approach and theoretical perspective proposed to enable the understanding of habitats as “multispecies landscapes” (see Glossary). Multispecies ethnography introduces a posthumanist perspective that deconstructs the “humanism” of landscapes. It recognizes that “other-than-humans” exist, and explores human social and cultural phenomena with respect to people’s relationships with other species through a network of interspecies encounters. Kirksey and Helmreich’s (2010) proposal for a “multispecies ethnography” has gained considerable support, as it allows broader manifestations of nonhuman organisms to appear alongside humans as animated beings (Baynes-Rock 2013; Lestel and Taylor 2013). Multispecies studies perceive nonhumans acting with “agency and intent” (see Glossary), while some definitions draw on understandings from Actor–Network Theory that considers agency as an effect rather than the product of subjective intentionality (Ogden *et al.* 2013 provide a detailed explanation) (Locke 2017). In this perspective, “creatures previously appearing on the margins of anthropology – as part of the landscape, as food for humans, as symbols – [are] pressed into the foreground of recent ethnographies” (Kirksey and Helmreich 2010, p. 545). Similar ideas have been described as “a more-than-human approach to ethnographic research” (Locke and Münster 2015, p. 1) and “an anthropology beyond the human” (Kohn 2013).

Viewing humans and nonhuman species as interacting organisms that shape and create ecosystems reflects the worldviews of many animist communities (Descola 1994; Ingold 2000, 2011; Kohn 2013). For some human groups, such as the Nyishi people of upland Arunachal Pradesh in northeast India, “animated beings” extend to include natural entities and supernatural beings as well as living organisms (Aisher 2007; Aisher and Damodaran 2016). Therefore, approaching primate conservation through a multispecies lens and understanding habitats as multispecies landscapes not only supports the conservation of wildlife for its intrinsic value, regardless of function or value to humans (Pearson 2016); it also promotes the cultural diversity of local communities. It acknowledges alternative realities that guide a conceptual shift toward environments being viewed and managed with respect to the ontologies of local people, which could improve the long-term outcomes of conservation initiatives (Keil 2016).

The idea that humans and nonhuman species shape environments through their interactions with each other is also explored in the biological sciences through niche construction (Barker and Odling-Smee 2014; Day *et al.* 2003; Odling-Smee *et al.* 2013) and through *natureculture* “contact zones”—terms adopted from the social sciences (Fuentes 2010a; Riley and Fuentes 2011) (see Glossary). In ethnoprimatology, Fuentes (2010a) employs the biological “niche construction model” and theory of “mutual ecologies” (Barker and Odling-Smee 2014; Odling-Smee *et al.* 2003) alongside Haraway’s (2008) “contact zones” (see Glossary) to describe the interface between tourists and long-tailed macaques (*Macaca fascicularis*) at temples in Bali. His description of “natureculture contact zones” (Fuentes 2012) recognizes that broad species characteristics as well as individual idiosyncracies are both the cause and outcome of the ways in which individuals act and interact. Sympatric primate species maintain their individuality, yet their destinies are united through historical events and embedded in shared environments (Haraway 1997, 2010). Fuentes argues that in doing so, the boundaries separating humans and macaques are broken down as the overlapping ecologies of these coexisting species generate coproduced niches. Ecological interactions are incorporated alongside social, historical, political, and economic drivers demonstrating that the inclusion of anthropological elements is core to primatological inquiry.

Ethnoprimatology deepens our understanding of human–primate coexistence by exploring overlapping ecologies at the human–primate interface, and integrating multispecies approaches with ethnoprimatology takes this concept a step further (Fuentes 2010a; Malone *et al.* 2014; Palmer and Malone 2018; Remis and Jost Robinson 2017). Combining ideas from multispecies ethnography (such as viewing environments as *multispecies landscapes*) with terminologies already applied in ethnoprimatology (such as the coexistence of sympatric species in shared ecological and social spaces) encourages researchers to revise the way they think and talk about environments and nonhuman species. This perspective helps deconstruct deep-seated preconceptions about the “humanism” of places and habitats and allows focus on the connections between multiple species (including people and primates) (Locke and Münster 2015). The case study that follows describes research carried out by three of our authors (K. J. Hockings, A. Frazão-Moreira, and H. E. Parathian) between January 2012 and November 2013 to explore coexistence between humans and other primates in Guinea-Bissau, West Africa. It illustrates how combined methods and theories from ethnoprimatology and multispecies ethnography can be applied through interdisciplinary research approaches to explore the connections between humans and primates sharing ecological and social spaces, and how this information can be used to inform conservation guidelines.

## A Cross-Disciplinary Understanding of Human–Chimpanzee Coexistence at Cantanhez National Park, Guinea-Bissau

**Fig. 1:**
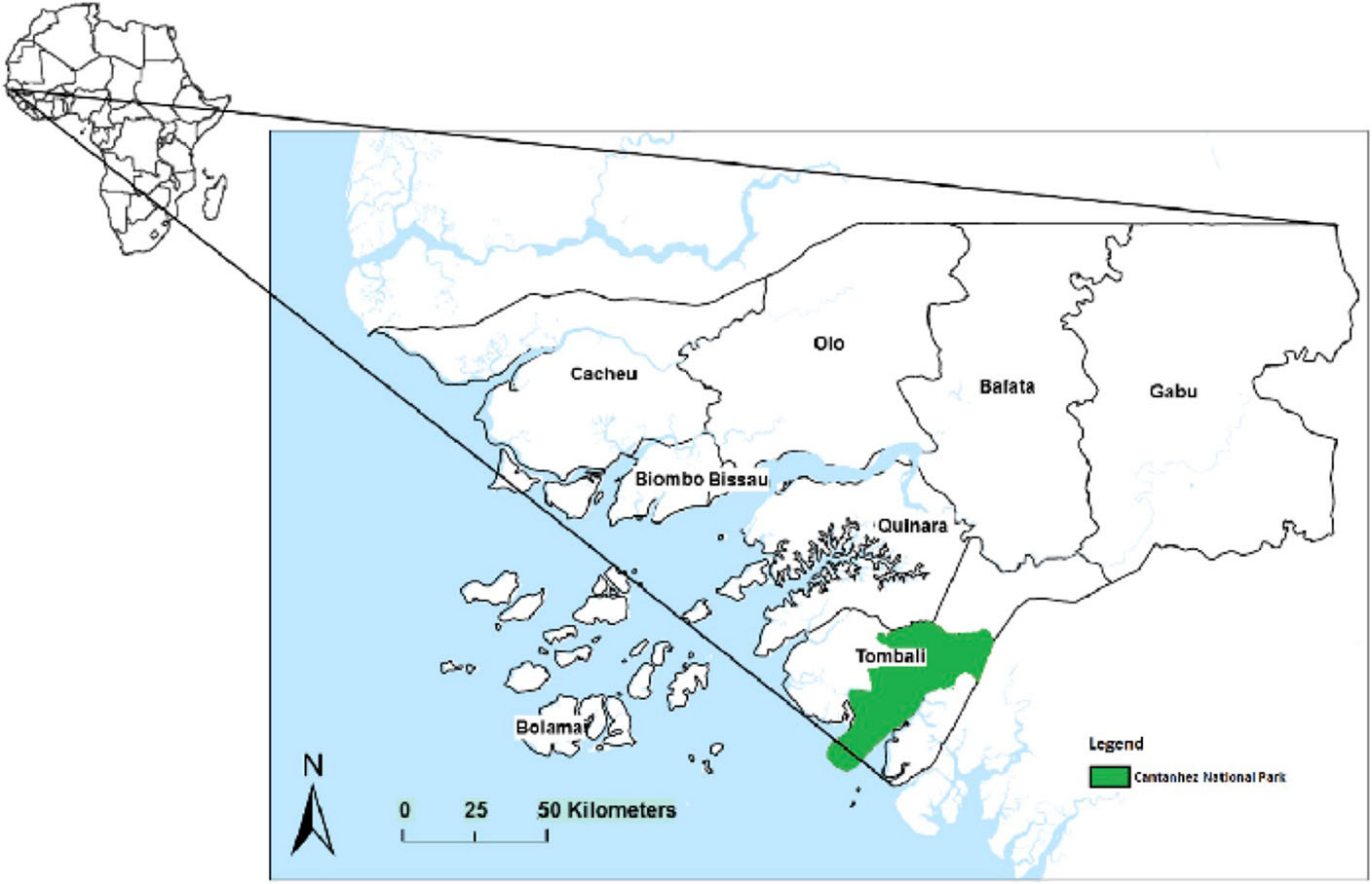
Map showing Cantanhez National Park in Guinea-Bissau, West Africa.

**Fig. 2:**
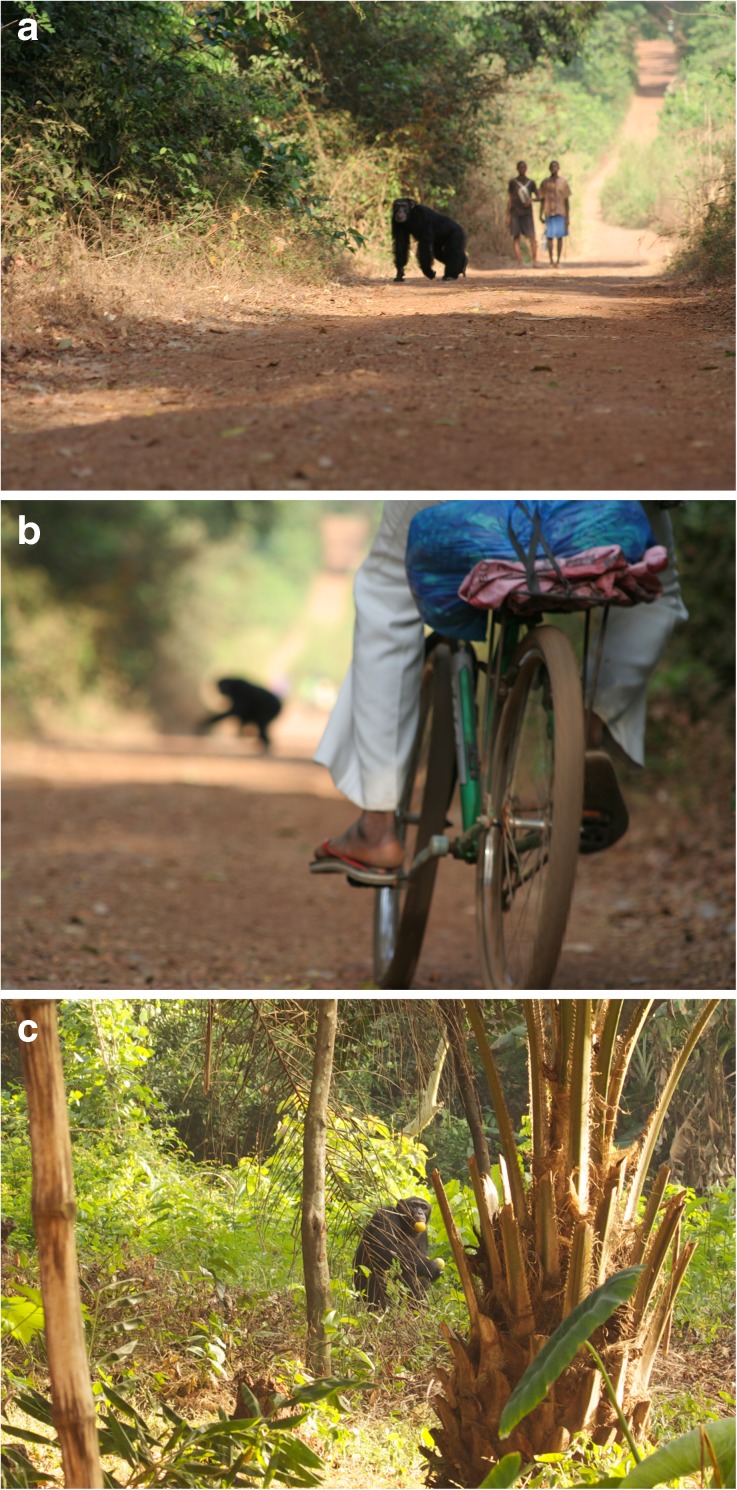
**a** Local people and chimpanzees encountering each other on a road in Cantanhez National Park (photo by K. Hockings). **b** A cyclist passing a chimpanzee that is crossing the road in Cantanhez National Park (photo by K. Hockings). **c** An adult male chimpanzee transporting cultivated oranges in an agricultural field next to the village (photo by J. Bessa).

Cantanhez National Park (CNP) is located in the southern Tombali administrative region of Cubucaré in Guinea-Bissau (Fig. 1). Covering an area of 1067 km^2^, the park is a mosaic of settlements, agricultural fields, subhumid and secondary forest, mangrove, and savanna. Six ethnic groups live within CNP with a total human population of *ca*. 22,500 individuals (Temudo 2009). Historically, all ethnic groups apart from the Balanta (who adopted Christianity alongside animism) were Islamized during the late nineteenth and early twentieth century. This led to the regional assimilation of Islamic and animist beliefs and practices (Frazão-Moreira 2009, 2010; Sousa *et al.* 2017). The Nalu ethnic group was among those people who were Islamized. The Nalu practice swidden agriculture and harvest wild resources for a range of uses, and their traditional practices link spirits (*irã*) and ancestors to local territory and Nalu homeland (see Frazão-Moreira 2009, 2016b). The forests of CNP are also inhabited by western chimpanzees whose range covers part of the protected area legally recognized as Nalu homeland (including the population of chimpanzees that were the focus of our study; Bessa *et al.* 2015; Hockings and Sousa 2012, 2013). As occurs elsewhere in tropical Africa (Hockings and McLennan 2016; McLennan and Hockings 2016), people and chimpanzees at CNP encounter each other frequently on roads, paths, in agricultural fields, and in the forest, and overlap in their use of wild and cultivated resources (Fig. 2a–c) (Hockings and Sousa 2012, 2013; Sousa 2009; Sousa and Frazão-Moreira 2010). Following the formation of CNP in 2008 the Nalu maintained ownership over part of the forest and have continued to play a role in its management, including the distribution of land to incoming settlers (Frazão-Moreira 2009, 2010). Therefore, our research focused on interactions between chimpanzees and Nalu people in particular. The known complexity of factors influencing the availability and management of resources in CNP, as well as limited data on overlapping habitat and resource use by people and chimpanzees, impelled us to design and implement a mixed-methods approach. We explored these dynamics from a multispecies perspective, combining ethnoprimatology with multispecies ethnography, which further integrates anthropological and biological approaches.

### Previous Studies in CNP

Previous ethnographic and botanical accounts among Nalu people have resulted in in-depth and insightful publications on indigenous plant use (Catarino *et al.* 2008; Frazão-Moreira 2009, 2010, 2016a, b), while ethnoprimatological studies have explored Nalu relationships with chimpanzees in CNP (Costa *et al.* 2017; Hockings and Sousa 2013; Sousa and Frazão-Moreira 2010; Sousa *et al.* 2014, 2017, 2018). These studies show that Nalu people have a syncretic Islamic-animist view toward animals, which combines the idea that “*dari i pekador*” (“the chimpanzee is human”) and the general belief that all nonhuman species have reputed access to resources in ancestral lands, with Muslim *aram*, which prevents the killing and eating of any animal with canine teeth, including primates. The Nalu recognize the similarities chimpanzees share with humans both physically and behaviorally (e.g., “*Dari* are like humans because they walk without putting their hands on the ground” and “They are like us. They use the same plants that we use”) (Sousa and Frazão-Moreira 2010). Other ethnographic accounts suggest Nalu people’s attitudes toward chimpanzees in CNP stem from an animist ontology which guides local beliefs that nonhuman species exist either as “true animals,” or some other animal form transformed by *irãs* (Sousa *et al.* 2017, 2018). This idea that humans and great apes shape-shift into each other’s physical forms is shared by people elsewhere in West and Central Africa (Giles-Vernick and Rupp 2006; Hockings *et al.* 2010; Köhler 2005; Leblan and Bricka 2013; Oishi 2013; Richards 1995). The underlying components of a pre-Islamic ontology combined with Muslim beliefs is key to understanding human–chimpanzee coexistence in CNP (Costa *et al.* 2017; Sousa and Frazão-Moreira 2010).

As well as processes of religious and cultural syncretism, local perceptions have evolved in CNP with conservation and ecotourism development supporting the protection of chimpanzees (Costa *et al.* 2017; Sousa *et al.* 2014, 2017, 2018). Despite strict beliefs that prevent chimpanzee hunting and consumption of their meat, according to some Nalu people conflicts between people and chimpanzees occurred in the past over highly valued agricultural resources (e.g., cultivated fruits and cash crops such as oranges and papaya). This reportedly led to potentially accidental killings of chimpanzees, where chimpanzees were shot at by local people to keep them away from crops (specifically during harvest and fruiting seasons) (Sousa and Frazão-Moreira 2010). However, since the active promotion of chimpanzee conservation and “ecotourism” by outside agencies and local NGOs, villagers claimed they no longer shot at chimpanzees for fear of retribution from the authorities (Sousa and Frazão-Moreira 2010). Other social science research suggests tensions exist between local people and NGOs, and that the sense of an urgent need to conserve wildlife in CNP—conveyed by National Park authorities and conservationists working in the region—is not always shared by local people (Sousa *et al.* 2018; Temudo 2012). For example, Temudo (2012) argues that outside agencies have constructed a need for conservation intervention in CNP based on inaccurate predictions (of the rate of deforestation, and the growth of human population densities) and the oversight of Nalu natural resource management institutions and practices, resulting in negative consequences for local people.

### Our Research Team and Field Study Approach

Our field research team comprised primatologists with broad experience in human–chimpanzee coexistence in Africa (K. J. Hockings, C. Sousa) as well as social scientists with long-term fieldwork experience among rural communities in Guinea-Bissau (A. Frazão-Moreira, H. E. Parathian), and three of our researchers had designed and implemented mixed-methods studies in previous research projects (K. J. Hockings, A. Frazão-Moreira, H. E. Parathian). Combining multispecies ethnography with ethnoprimatology, we began by carrying out an in-depth ethnography of Nalu beliefs and practices associated with the forest and primates to examine the connections between Nalu people and chimpanzees in CNP. Qualitative social data added context to quantitative findings and provided information about current local attitudes toward chimpanzees. This provided a strong starting point from where we were able to explore the influence of individual species behaviors, and the impact of interspecies interactions, on the local landscape from a multispecies perspective. To further explore human–chimpanzee coexistence and resource-sharing at a social, historical, and ecological level, and the influence of local Nalu cultural and religious beliefs on these dynamics, we employed tools from ethnoprimatology and ethnobotany. Over eleven months the social science researchers (H. E. Parathian and A. Frazão-Moreira) and biological science researchers (K. J. Hockings, C. Sousa, and J. Bessa) conducted complementary research on the use of wild and cultivated resources by sympatric humans and chimpanzees using comparative methods. We collected quantitative data (through direct observation, feeding traces, and fecal analysis) to determine which plants and plant parts were consumed by chimpanzees. We compared these data with quantitative data on human plant use (collected through participant observation, semistructured interviews, and all-occurrence sampling). Finally, we carried out spatial mapping to identify overlapping areas where humans and chimpanzees used plants, providing a visual representation of the CNP forest as a multispecies landscape shared and shaped by sympatric species.

Openness, trust, and good communication among our field team were key to the smooth running of our study. Project planning took place with input from our biological and social science researchers to limit misunderstandings and prevent disciplinary disputes between researchers from different academic fields. We held regular meetings to share data and discuss the progress of each component of the research. All members of the team were motivated to work together despite differences in disciplinary training, because of a common concern for conservation, alongside enthusiasm for the research proposal, and mutual respect for the value of each other’s work. While the primatologists were concerned mainly with understanding the behavior and ecology of chimpanzees, and how these are influenced by people (data important for chimpanzee conservation in CNP), they recognized the value of local concepts of forest management, and the importance of understanding plant use overlap between villagers and chimpanzees to predict the sustainability of their interactions in this shared environment. For the social scientists, their motivation was guided by an interest in supporting indigenous advocacy and establishing the rights of local people to access natural resources in CNP. An integral part of supporting people’s access to resources involved exploring local environmental perceptions, including understanding people’s representations of wildlife. For the Nalu, ideas about chimpanzees and plant use form a central part of explaining their perspectives of and attitudes toward wildlife, and our research team appreciated the interdisciplinary focus of the study was a vital component to interpreting this accurately.

### Summary of Findings

Our study showed that Nalu people and chimpanzees “meet” frequently in CNP and overlap extensively in their use of wild resources, including important chimpanzee foods such as oil palm (*Elaeis guineensis*), velvet tamarind (*Dialium guineensis*), and saba (*Saba senegalensis*) (Hockings *et al. unpubl. data*). The regular overlap of land and resource use between these sympatric species has led to a degree of mutual tolerance. The chimpanzees have adapted their foraging behavior in response to changes in human foraging and cultivating patterns; for example, the chimpanzees frequently consume cultivated foods (Bessa *et al.* 2015). For their part, Nalu people have moved away from using rifles and some have adopted alternative strategies and precautions to prevent crop damage and reduce negative interactions with chimpanzees, with some people reporting that they intentionally do not cut important chimpanzee wild food species. When people encounter chimpanzees on roads, in their gardens, or near their homes, they generally respond calmly to their presence. Only on occasions when chimpanzees are in close proximity to children or women are people likely to shout and throw objects such as sticks in an effort to deter chimpanzees from approaching. Such behaviors reportedly can incite retaliatory aggression from chimpanzees elsewhere (McLennan and Hockings 2016); however, harmful behavior by chimpanzees toward people has rarely been reported at CNP (Hockings and Sousa 2013; Sousa *et al.* 2017). Moreover, Nalu people coexist with chimpanzees with relatively low levels of hostility as compared to interactions in some other regions (e.g., parts of western Uganda where chimpanzee habitat has been converted to agricultural land and spatial overlap with villagers is exceptionally high: Hockings and McLennan 2016; McLennan 2008). This relative tolerance of Nalu people toward chimpanzees arises from complex cultural, economic, and ecological factors that may be resource specific. For example, our findings show that chimpanzees are not considered to cause significant damage to the main cash crop, cashew (*Anacardium occidentale*), as chimpanzees feed only on the cashew pseudofruit, leaving the economically valuable cashew nut undamaged. According to Nalu people, chimpanzees leave the nuts in piles, thus helping them with the cashew nut harvest; the cashew fruit consumed by the chimpanzees in the process is regarded as fair payoff in exchange (Bessa *et al.* 2015; Hockings and Sousa 2012).

While local people are tolerant of chimpanzees, these interactions may not be quite as straightforward as they first appear because of associations with sorcery, where chimpanzees are incorporated into local cosmologies via their association with witchcraft. Other studies show that the complexity of local people’s relationships with chimpanzees (and some other wild animal species) has consequential, sometimes unforeseen outcomes for conservation. For example, Sousa *et al.* (2017, 2018) reported that the stories and descriptions about chimpanzees shared by local people with outsiders do not always represent their true sentiments about these great apes or certain local conservation initiatives. Despite no attacks being reported during our research period, local descriptions of chimpanzee attacks on people recorded by Sousa and colleagues distinguish between attacks by “clean” animals and attacks by “unclean” or “shape-shifted” individuals. Attacks by “unclean” chimpanzees, i.e., sorcerers who practice shape-shifting and have taken on the appearance of chimpanzees to further their own interests, are associated with situations of perceived abuse of power and expressions of greed. Attacks on persons by “clean” animals, i.e., chimpanzees responding to an antagonistic situation/stimulus, are interpreted as animals defending themselves or their group members against a tangible threat, and therefore are regarded as “natural” and a reasonable response on the part of the animal. Therefore, under certain circumstances local people perceive chimpanzees as akin to humans who commit socially or culturally harmful behaviors to others. In recent years this analogy has been extended to include the abuse of power that sometimes exists between NGOs and local people, suggesting a degree of unease among the local population, directed at conservation more generally rather than toward the chimpanzees themselves (Sousa *et al.* 2017, 2018).

Furthermore, while some studies at CNP indicate a degree of resistance among local people toward conservation initiatives (Sousa *et al.* 2017, 2018; Temudo 2012), our findings suggest that cultural and religious beliefs alongside economic and ecological factors result in conservation outcomes that protect chimpanzees in CNP to some extent. We held a participatory workshop in December 2016 to share research findings and consult with local people on chimpanzee conservation. Participants, including young people, women, men, male and female leaders, and guides working for the National Park, not only indicated tolerance toward chimpanzees feeding on plant species that are highly valued by people, but also suggested a general acceptance and acknowledgment over conservation concerns among researchers and NGOs developing chimpanzee conservation in the region. These findings, along with our data on human and chimpanzee plant use in CNP, are currently being used to inform decisions going forward for chimpanzee conservation at a local and national level in Guinea-Bissau.

### Summary

Merging various methodologies enabled us to advance beyond more typical ethnoprimatology techniques (discussed previously) and adopt a multispecies approach, viewing CNP from a Nalu perspective and acknowledging chimpanzees as compatriots living alongside them with ancestral and historical links to Nalu territory (*cf.* Jost Robinson and Remis 2014; Remis and Jost Robinson 2017). This approach allowed us to begin to explore the local landscape and the sustainability of human–chimpanzee coexistence in CNP, giving equal weight to both species, within changing environmental, social, and economic conditions. We have shown how humans and chimpanzees are constituted in and by their relations to each other where they meet and “mingle” (Haraway 2008, 2010), sharing habitat and resources. As human populations expand, in part due to migration from nearby countries, pressure on key resources such as land and certain wild plants will increase in CNP, which may again cause changes to human–chimpanzee relationships in response to new conditions, as seen elsewhere (e.g., in Uganda: McLennan and Hill 2012, and in Central African Republic: Jost Robinson and Remis 2014). Understanding human–primate coexistence alongside different interest groups’ agendas and priorities becomes critical if environmental and conservation policies are to be effective and keep pace with these changes. Studies that explore advanced approaches in ethnoprimatology and encourage mixed-methods research, such as ours, provide new possibilities for locally appropriate conservation in shared landscapes.

## Conclusions: Mainstreaming the Multispecies Approach in Primate Conservation

As major niche constructors, humans have had a consequential impact on the lives of other primates (Fuentes 2010a), just as living with primates has likely characterized much of our own evolutionary history (Riley 2006; Tutin and Oslisly 1995) and continues to do so. Integrating social science with conservation science approaches is crucial to understanding when and under what conditions human–primate sympatry is sustainable (McLennan *et al.* 2017). We have described how the multispecies approach is part of a broader aim by social scientists to overcome anthropocentrism in the study of human–nature interactions by theoretically integrating relational perspectives into Western science (Locke and Münster 2015). Continuing to apply a multispecies lens to ethnoprimatological research and maintaining the shift in focus from a *conflict* to *coexistence* narrative has the potential to produce more positive long-term outcomes for people and wildlife (Fuentes and Hockings 2010; Fuentes *et al.* 2016; Hardin and Remis 2006; Hill and Wallace 2012; McLennan *et al.* 2017). This entails bridging theory between the biological and social sciences and integrating our efforts to ensure productive conservation discourse for the benefit of both people and wildlife. We have shown that a more cohesive study of human–primate worlds can inform our understanding about interspecies interactions and multispecies landscapes. Our case study presents one example of how promoting engagement between the social sciences and disciplines traditionally grounded in the biological sciences can further develop the ethnoprimatology approach to deepen our understanding of environments from a multispecies perspective. Supporting a perceptual shift toward interdisciplinary research that combines multispecies ethnography with ethnoprimatology will further advance the development of these ideas, helping establish a more integrated and holistic biological and cultural conservation.

Working to improve interdisciplinary collaboration presents a challenge for academics and practitioners alike, but may be crucial to avert the extirpation of primates among other wildlife across the globe (Estrada *et al.* 2017). The true mainstreaming of social science in conservation needs visionary leadership and a dramatic change in organizational behavior (Bennett *et al.* 2017a, b; Mascia *et al.* 2003), potentially including the reorganizing of academic communities, funding, and institutions as a way of increasing avenues for collaboration between the different sciences (Palsson *et al.* 2013; Teel *et al.* 2018). This requires building social science capacity into conservation agencies, promoting engagement between the social sciences and disciplines traditionally grounded in the biological sciences including primatology, overcoming the associated political challenges that cross-disciplinary engagement often incurs, and willingness among social scientists to engage with biological scientists and share knowledge, insights and recommendations in an open and constructive way (Palsson *et al.* 2013; Redford 2011). Methodological expertise and skilled practice are not easily acquired, providing a further incentive for cross-disciplinary collaboration. If done well, this could produce positive results in the field of primate conservation. Primate researchers must rise to the challenge and become skilled at bridging disciplinary boundaries to provide a better understanding of the complexity in which conservation occurs (Fuentes and Hockings 2010; Fuentes *et al.* 2016; Riley and Fuentes 2011; Setchell *et al.* 2017). As Castree *et al.* (2014, p. 763) write, “interdisciplinary dialogue [we suggest] should engender plural representations of Earth’s present and future that are reflective of divergent human values and aspirations.”

### Data Availability

The datasets analyzed during the current study are available from the corresponding author on reasonable request.

## Glossary

Academic culture: The totality of socially transmitted behaviors, beliefs, institutions, and other products of human work and thought, with respect to a particular field, subject, or mode of expression (Morris 1969; Sutherland 1998).
Commonly Used Terminology in Biological Anthropology: ᅟ
Biological anthropology: “Biological” (or “physical”) anthropology is concerned with the biological and behavioral aspects of humans, nonhuman primates, and their extinct hominin ancestors. It provides a biological perspective to the systematic study of primates. As a subdiscipline of anthropology, biological anthropology is divided into several branches united in their common application of evolutionary theory to understanding human morphology and behavior, such as paleoanthropology and primatology (Fuentes 2010b).
Coexisting/sympatric species: Species that occur at the same time period and in the same place and can potentially interact (Cormier 2010; Fuentes and Wolfe 2002; Wheatley 1999).
Ethnoprimatology: Interdisciplinary study developed by primatologists, combining primatological and ethnographic practice to examine the multifarious interactions and interfaces between humans and nonhuman primates living in integrated and shared ecological and social spaces (Fuentes 2012; Hockings *et al.* 2015; Sponsel 1997). The goal of many ethnoprimatology studies is to understand the perceptions of local people and engage with their needs to enhance primate conservation and ensure the longevity of conservation projects (Lee 2010; Wheatley 1999). It adopts a mosaic of approaches that develops and reshapes the ways in which humans position themselves relative to human-dominated landscapes and ecologies (Dore *et al.* 2017).
Human–primate interface: Description of overlapping ecologies of human–nonhuman primate communities, viewing humans as literal and figurative kin to other primates. This term plays a core linking role in ethnoprimatology, between anthropology and primatology studies (Fuentes 2012; Leblan 2013; Wheatley 1999).
Human–wildlife conflict: Negative interactions between humans and wildlife where one or both species suffers as a consequence (Woodroffe *et al.* 2005). Researchers are increasingly moving away from this term when referring to scenarios in which wildlife impact on people’s livelihoods, security, or personal safety. Its use obscures the fact that these “conflicts” often stem from differential values, needs, priorities, and power relations between the human groups concerned (Hill 2015, 2017; Hill *et al.* 2017; Redpath *et al.* 2013).
Human–wildlife interactions: Traditionally understood in biology as people and wildlife sharing landscapes and resources, ranging from being beneficial or harmful to one species or the other. In ethnoprimatology human–wildlife interactions are increasingly understood as being dynamic and bidirectional (Humle and Hill 2016; Lee 2010; Wheatley 1999).
Niche construction: The creation and destruction of environments by organisms, and their interactions with other individuals (comprising synergistic interactions between organisms and environments). Through these processes the selective pressures that impact organisms are shaped (Barker and Odling-Smee 2014; Day *et al.* 2003; Odling-Smee *et al.* 2003, 2013). Specifically, in terms of anthropology, this perspective suggests ways in which behavioral and symbolic systems construct and interact with social and ecological niches and how, in turn, these systems interact with genetic systems (Fuentes 2010a).
Commonly Used Terminology in Social/Cultural Anthropology: ᅟ
Agency/intent: Having an independent capability or ability to act on one’s will. The capacity of individuals to make their own free choices and their reasons for acting are affected by cognitive belief structures that form through experiences, and societal/individual perceptions. This contrasts with structure, which describes factors of influence (such as social class, religion, gender, ethnicity, ability, customs, etc.) that determine or limit an agent and his or her decisions (Barker 2005).
Companion species: A term used to describe the historical emergence of wild and domestic animals in human lives. Nonhuman species are recognized as individuals who are part of historical relationships with individual people and human communities (Baynes-Rock 2013; Haraway 2010, 2016).
Contact zone: Places where humans and nonhumans share physiological, ecological, and social spaces across scales of ecological intersection. It signifies how subjects are constituted in and by their relations to each other (Haraway 2008).
Interspecies mingling: A term used to describe the mixing or bringing together of different human–nonhuman species without the individual’s fundamental loss of identity (Haraway 2010).
More-than-human /Anthropology beyond the human: These terms introduce perspectives that extend anthropology to a posthumanist inquiry through the application of multispecies ethnographies (Kirksey and Helmreich 2010; Kohn 2013).
Multispecies ethnography: A methodological approach and theoretical perspective rooted in anthropology that deconstructs the “humanism” of landscapes (Kirksey and Helmreich 2010) and enables the understanding of habitats as “multispecies landscapes.” Multispecies ethnography is concerned with the connections between humans and other life forms (which also have agency and intent or whose actions are the result of agency as an effect, rather than as the product of subjective intentionality; see earlier). It acknowledges that the human condition cannot be understood in isolation from nonhuman species (Locke 2017; Ogden *et al.* 2013).
Multispecies lens: Examining human–nonhuman interactions where humans are viewed as one of several organisms that shape, create, and form an integral part of their environment, because of engagements and interactions with nonhumans. In this context environments are viewed as “multispecies landscapes” through a “multispecies lens” (Aisher and Damodaran 2016).
Social/Cultural Anthropology: The fields of “social” and “cultural” anthropology overlap to a considerable extent. Broadly, the term “cultural anthropology” relates to an approach prominent in the French tradition and the United States. It stresses the coherence of human cultures, including their rules of behavior, language, material creations, and ideas about the world. “Social anthropology,” developed in the United Kingdom during the early years of the twentieth century, is a scientific discipline with an emphasis on human social institutions, their interrelationships, and the organizing principles of social and cultural life (Erickson 2011).
